# Book Review: Exploring Student Loneliness in Higher Education: A Discursive Psychology Approach

**DOI:** 10.3389/fpsyg.2021.687924

**Published:** 2021-05-13

**Authors:** Hao Liu

**Affiliations:** Department of English, School of Languages and Culture, Tianjin University of Technology, Tianjin, China

**Keywords:** student loneliness, higher education, discursive psychology, loneliness disclosure, constraints, social credentials, temporal contrasts

Loneliness is a picture highly present but rarely visible in education. In the seminal work of Weiss (1973), loneliness is considered as a “gnawing, chronic disease.” Previous studies indicated that some connections can be drawn between loneliness and issues such as student retention, student engagement, and student well-being (Wilcox et al., 2005). The reliance on subjective interpretations, however, makes the study of loneliness a particularly difficult topic. The linguistic turn in emotion studies has led to a call for more attention to look into emotion talks in everyday contexts. This book under review reports the finding by presenting several themed Discursive Psychological analyses of student interview data on loneliness. It provides insightfully sensitive and perceptive analysis with far-reaching implications for future studies.

The book consists of six chapters. Chapter 1 provides an overview of the study of loneliness and Discursive Psychology (DR) and sets the scene for the following chapters. Chapter 2 relates to the disclosures of loneliness occasioned within the student interviews. Chapter 3 explores students' personal, structural and cultural constraints, and the factors accounting for their feelings of loneliness. Chapter 4 concerns social dispositions and social networks invoked by the students to mitigate their loneliness disclosures. Chapter 5 addresses temporal contrasts and exemplifies several discursive devices utilized by the students to attend to impression management. Chapter 6, a summary of the book, offers suggestions for future lines of enquiry in the study of loneliness.

This book stands out because it introduces DP approach to analyze student loneliness in higher education. It demonstrates that both interactional detail (i.e., the words of what one says) and paralinguistic features such as laughter, prosody, and hesitations contribute to disclosures of loneliness. Its merit lies in the following four aspects. First, it is a topical book focusing on the detail of narrated experience of student loneliness. It is often difficult to identify a well-being issue with a student until it has reached a visibly problematic point. The book traces the evolution of loneliness from Weiss's seminal study in 1973 through to present-day psychological and sociological concerns with the topic, and specifically highlights the accounts of the students who have felt loneliness.

Secondly, the book explores student loneliness from the DP perspective, reflecting a trend of linguistic turn in the emotion studies. As a theoretical and analytical approach to discourse, DP treats talk and text as an object of study in itself, and takes psychological concepts as socially managed and consequential in interaction (Wiggins, 2017). In the book, loneliness is viewed as “a physiological response which individuals imperfectly try to describe and account for” (p.178), and the conceptualizations of loneliness experiences are believed to be mediated through specific social interaction. To make sense of the complex and highly contextual process, four new assumptions are formed to understand the deployment of lonely feelings in interaction.

Thirdly, the book foregrounds semi-structured interview, a more linguistically oriented methodology, in the analysis of loneliness. The prior mainstream psychological research on loneliness has drawn upon instruments such as questionnaires and scales to investigate loneliness within university settings. Those who get the higher scores are considered more lonely. The standard to evaluate students' loneliness feelings and physiological experiences, however, should not be measured and reduced to a number based on limited isolated reflections. Rather, it should be best studied as the product of social interaction and meaning-making practices.

Finally, this book provides contextually bound inference to loneliness. For example, Chapter 4 sees constraints as discursive resources in conversation. It highlights that student interviewees make relevant discursive forms and membership categories as constraints during their disclosures of loneliness. Operationally, it proposes a typological distinction between personal, structural, and cultural constraints. Another strong point is its co-constructed nature of discursive actions. Rather than record only student interviewee's words and miss interviewer's words, the book provides sufficient context in the transcripts, and locates the answers and questions within broader sequence of the interview itself. Additionally, dynamic analysis is used to probe into student loneliness, and this dynamism is facilitative to record the temporal narratives and to explore how students work up their dual identities at different points in time.

Nevertheless, there remain some aspects for this book to be improved in its future edition. First, it would be more thorough if the book could include empirical studies and narratives of university students beyond the UK context, as voices from those students may introduce immensely varied landscapes of loneliness across languages and cultures. Second, readers may benefit more from it if it could offer a more comprehensive solution, preferably a model, to this long-neglected challenge facing students in higher education today.

Overall, Oakley's book is an important contribution to research on student loneliness. It brings psychological and linguistic insights to bear on our understanding of the way loneliness are expressed and experienced. The book further provides a new Discursive Psychological framework that can be referred to by researchers in the analysis of loneliness talks. The underlying assumptions and principles are suitable for all stakeholders to further study students' discursive strategies around loneliness. It is highly recommended to anybody interested in learning about and further exploring student loneliness.

## Author Contributions

The author confirms being the sole contributor of this work and has approved it for publication.

## Conflict of Interest

The author declares that the research was conducted in the absence of any commercial or financial relationships that could be construed as a potential conflict of interest.
